# Human exhaled air can efficiently support *in vitro* maturation of porcine oocytes and subsequent early embryonic development

**DOI:** 10.21451/1984-3143-AR2017-0027

**Published:** 2018-08-16

**Authors:** Zubing Cao, Di Gao, Tengteng Xu, Xu Tong, Yiqing Wang, Yunsheng Li, Fugui Fang, Jianping Ding, Xiaorong Zhang, Yunhai Zhang

**Affiliations:** Anhui Provincial Laboratory of Local Livestock and Poultry, Genetical Resource Conservation and Breeding, College of Animal Science and Technology, , , .

**Keywords:** human exhaled air, pig, oocyte maturation, somatic cell nuclear transfer, early embryo

## Abstract

Air phase is an indispensable environmental factor affecting oocyte maturation and early embryo development. Human exhaled air was previously proved to be a reliable and inexpensive atmosphere that sustains normal early development of mouse and bovine embryos. However, whether human exhaled air can support *in vitro* maturation (IVM) of porcine oocytes is not yet known. To evaluate the feasibility of maturing oocytes in human exhaled air, we examined oocyte morphology, *BMP15* expression, nuclear and cytoplasmic maturation. We found that cumulus expansion status, expression levels of *BMP15* important for cumulus expansion and the rate of first polar body emission were similar among human exhaled air, 5% O_2_ or 20% O_2_ in air after IVM of 44 h. Furthermore, the percentage of metaphase II (MII) oocytes showing normal cortical and sub-membranous localization of cortical granules and diffused mitochondrial distribution patterns is comparable among groups. The cleavage, blastocyst rate and total cell number were not apparently different for parthenogenetic activated and somatic cloned embryos derived from MII oocytes matured in three air phases, suggesting oocytes matured in human exhaled air obtain normal developmental competence. Taken together, human exhaled air can efficiently support *in vitro* maturation of porcine oocytes and subsequent early embryonic development.

## Introduction

The faithful achievement of mammalian oocyte maturation is an essential prerequisite to carry out the robust preimplantation embryo development ( Gilchrist and Thompson, 2007 ; Coticchio *et al.* 2015 ). Oocyte maturation mainly consists of two key biological events involving a set of complex nuclear and cytoplasmic changes that determine oocyte quality ( Sun and Nagai, 2003 ; Krisher, 2004 ; Wang and Sun, 2007 ). At present, the external culture conditions of oocyte maturation *in vitro* are not yet optimal compared with naturally physiological environment in the reproductive tract ( Fischer and Bavister, 1993 ; Roberts *et al.*, 2002 ). This suboptimal culture environment may induce many detrimental roles in oocytes *in vitro* maturation and subsequent early embryonic development. Indeed, previous studies indicated that abnormal meiotic maturation could lead to a series of developmental defects involving meiotic progression arrest, aneuploidy in eggs and embryos, failure of pronucleus formation and even mitotic chaos in early embryonic development ( Howe and FitzHarris, 2013 ; Hu *et al.*, 2015 ; MacLennan *et al.*, 2015 ). In addition, incomplete cytoplasmic maturation including abnormal relocalization of cortical granules and mitochondria, and spatio-temporally translational failure of maternal mRNA, could cause dysfunctional epigenetic reprogramming and mitotic events important for fertilization, embryonic genome activation and blastocyst formation ( Pocar *et al.*, 2001 ; Dumollard *et al.*, 2007 ; Watson 2007 ; Chen *et al.*, 2013 ; Huan *et al.*, 2015 ). To date, accumulating evidence revealed that the developmental competency (also termed oocyte quality) of the resulting matured oocytes mostly depends on external culture environment during oocyte maturation *in vitro* ( Lonergan *et al.*, 2003 ; Wrenzycki and Stinshoff, 2013 ). Therefore, a stable and reliable incubation system for oocyte maturation *in vitro* is very important and deserves plenty of attention. In fact, it is discovered that culture conditions during *in vitro* maturation of oocyte encompass many facets, for example, compositions of culture medium, temperature, humidity, carbon dioxide and oxygen concentrations ( Bavister and Poole, 2005 ; Iwamoto *et al.*, 2005 ; Park *et al.*, 2005 ; Swain, 2010 ; Wrenzycki and Stinshoff, 2013 ). The majority of studies focuses on the optimization of composition of culture medium used for oocyte maturation *in vitro*, however, other environmental factors affecting oocyte maturation, such as culture air phase, also need to be examined.

So far, air phases commonly utilized for oocyte maturation *in vitro* are mainly separated into two categories involving 5% CO_2_,20% O_2_,75% N_ 2_ (high oxygen tension; Bavister, 1995 ) and 5% CO_2_,5% O_2_,95% N_2_ (low oxygen tension) ( Adam *et al.*, 2004 ; Kang *et al.* 2012 ). There are some disadvantages for *in vitro* maturation of oocytes using these two air phases. First, expensive standard incubator must be purchased and used to maintain the correct concentration of commercially available mixed gas stored in the cylinder. Second, consumption and cost of mixed air is also extremely expensive. Third, internal culture environment including temperature, humidity and CO_2_ equilibrium are often perturbed due to frequent openings of the incubator door which could result in suboptimal oocyte maturation and subsequent embryo development. Fourth, it is very difficult and dangerous for long-distance transportation of these two incubation apparatuses and is also not convenient to utilize them under field conditions, especially in animal farms. Thus, a simple, cost-effective, stable and reliable incubation system is desired to meet the need of oocyte maturation in the remote labs and animal farms. Two earlier studies demonstrated that mouse and bovine embryos cultured *in vitro* in the aluminium bag inflated with human exhaled air consisting of 4% CO_2_,16-17% O_2_,and 79-80% N_2_ were able to develop to blastocyst stage ( Tarkowski and Wroblewska, 1967 ; Vajta, 1997). However, whether this incubation system using human exhaled air could be employed to mature *in vitro* oocytes remains to be known. In this study, we used porcine oocytes as a model to investigate whether human exhaled air could support the normal nuclear and cytoplasmic maturation of porcine oocytes as well as subsequent early embryonic development.

## Materials and Methods

### Ethics statement

All animal experiments were conducted according to the Institutional Animal Care and Use Committee guidelines at Anhui Agricultural University.

### Chemicals and antibodies

All chemicals were purchased from Sigma (St Louis, MO) unless otherwise stated. MitoTracker Red CMXRos was purchased from Invitrogen (Cat. No: M7510).

### Preparation of human exhaled air incubation system

Airtight aluminium bag was sterilized by 75% alcohol in PBS and then these bags were naturally dried at room temperature. Four-well plates including porcine cumulus-oocyte complexes (COCs) were placed into the sterilized aluminium bag and then experimenter sealed the aluminium bag by sealing machine. A plastic tube equipped with 18-gauge syringe needle was used to connect bag with experimenters. Subsequently, healthy researchers started to exhale air into bag. Experimenter again sealed the bag to ensure the complete airtightness when it was inflated with appropriate exhaled air. Finally, the bag was placed into a constant temperature incubator without the inflation of commercial mixture gas and incubated for 44 h to allow the oocytes maturation. At the same time, the aluminium bag system was also used to culture COCs of the 5% CO_2_/5% O_2_ and 5% CO_2_/20% O_2_ groups.

### Oocyte in vitro maturation (IVM)

This experiment was performed as described previously ( Cao *et al.*, 2014 ). Briefly, ovaries from prepubertal gilts were collected at a local slaughterhouse and transported to the laboratory at 28ºC-35ºC in physiological saline solution containing penicillin (0.2 IU/mL) and streptomycin sulfate (0.2 IU/mL). The ovaries were washed in saline and the ovarian follicles from 3 to 6 mm in diameter were aspirated using a sterile 10 mL syringe with an 18-gauge needle attached. The aspirated follicular fluid was slowly injected into a preincubated 15 mL centrifuge tube to sediment the cumulus-oocyte complexes (COCs). The COCs with more than three layers of cumulus cells and homogeneous ooplasm were selected under a stereomicroscope. *In vitro* maturation (IVM) medium (TCM-199 supplemented with 15% FBS, 10 ng/mL EGF, 10% porcine follicular fluid, 10 IU/mL of eCG, 5 IU/mL of hCG, 0.8 mM L-glutamine and 0.05 mg/mL gentamicin) was pre-equilibrated overnight at 38.5ºC, human exhaled air, 5% CO_2_/5% O_2_ and 5% CO_2_/20% O_ 2_ in air, respectively. Subsequently, 50 of the COCs were washed and transferred in 4-well plate containing 400 μL IVM medium and the bags filled with three different gases were simultaneously put in a constant temperature incubator with 38.5ºC for 42-44 h. The COCs were then treated with DPBS without Ca^2+^and Mg^2+^ (Gibco, Grand Isle, NY) containing 1 mg/mL hyaluronidase to remove the surrounding cumulus cells. Finally, oocytes with clear perivitelline spaces, intact cell membranes, and extruded the first polar body (pb1) were selected for subsequent experiment.

### Parthenogenetic activation

Oocytes with first polar body emission were activated parthenogenetically by two pulses of direct current (1.56 kV/cm for 80 ms) in activation medium (280 mM mannitol, 0.1 mM CaCl_ 2_, and 0.1 mM MgCl_2_). Subsequently, embryos were washed in PZM-3 three times, followed by 4 h of incubation in the chemically assisted activation medium (PZM-3 supplemented with 10 μg/mL cycloheximide and 10 μg/mL cytochalasin B) covered by paraffin oil. Embryos were then washed three times with PZM-3 medium and cultured in fresh PZM-3 medium at 38.5°C, 5% CO_2_ and 95% air with saturated humidity.

### Preparation of donor cells

Landrace fetus in 35 days old was recovered and rinsed three times with PBS. The left tissues after removing head, intestine, liver, heart and limbs were cut into small pieces and incubated in fetal bovine serum (FBS). The tissue blocks were evenly smeared in a dish and cultured upside down at 37°C, 5% CO_2_ and saturated humidity. After 8 h of incubation, fibroblast cells were transferred into the standard cell culture medium (FBS supplemented with 85% DMEM, 0.1 mM NEAA, and 0.05 mM L-glutamine). Fetal fibroblast cells were dissociated and passaged until 90% confluence. Fetal fibroblast cells with 4th-8th generations were used as donor cells for nuclear transfer.

### Somatic cell nuclear transfer (SCNT)

SCNT was performed as described previously ( Cao *et al.*, 2015 ). Briefly, denuded MII oocytes were enucleated in manipulation medium (TCM199 supplemented with 2% FBS and 7.5 μg/mL cytochalasin B) through removing the first polar body and a small amount of the surrounding cytoplasm containing spindle using a 15–20 mm beveled glass pipette. Donor cells were injected into the enucleated oocyte to generate reconstructed couplets. Electric pulse was applied to the couplets in fusion medium, then immediately placed in PZM-3 medium. After 30 min of incubation, fused embryos were further incubated for 4 h in chemically assisted activation medium at 38.5°C and 5% CO_2_ with saturated humidity. Finally, embryos were washed three times using fresh PZM-3 medium and cultured in four-well plates containing PZM-3 medium at 38.5°C and 5% CO_2_ in humidified air.

### Evaluation of cortical granules distribution

The zona pellucida of MII oocytes was removed in 0.5% pronase solution, followed by washing in DPBS containing 0.3% BSA three times. Oocytes were fixed in 4% paraformaldehyde in DPBS for 30 min, followed by washing in DPBS supplemented with 0.3% BSA and 10 mM glycine. Oocytes were subsequently permeabilized in 0.1% Triton X-100 for 5 min at room temperature, followed by washing in DPBS containing 0.3% BSA two times. Oocytes were then labeled with 100 μg/mL FITC-conjugated peanut agglutinin (Sigma, L7381) in DPBS for 30 min in a dark chamber. Finally, the oocytes were washed three times in DPBS containing 0.3% BSA and 0.01% Triton X-100. DNA was labeled after staining with 10 μg/mL PI in DPBS for 10 min, followed by washing three times in DPBS, and then mounted on glass slides. Oocytes were imaged under an epifluorescence microscope (Olympus, IX71, Japan). Oocytes omitting the primary antibody were used as negative controls to examine the specificity of the reaction.

### Assessment of mitochondria distribution

1 mM stock solution of MitoTracker Red CMXRos was prepared in DMSO and stored at -20°C. Denuded MII oocytes were incubated for 30 min in IVM medium supplemented with 0.5 μM/L MitoTracker Red CMXRos at 38.5°C and 5% CO_2_ in humidified air. Oocytes were then washed three times in DPBS containing 0.3% BSA. Subsequently, oocytes were fixed in in 4% paraformaldehyde in DPBS for 30 min, followed by washing in DPBS supplemented with 0.3% BSA and 0.01% Triton X-100. Finally, the chromosomes of oocytes were labeled with 10 μg/mL Hoechst33342 in DPBS for 10 min, followed by washing three times in DPBS, and then mounted on glass slides. Oocytes were imaged under an epifluorescence microscope (Olympus, IX71, Japan).

### Quantitative real-time PCR (qRT-PCR)

Total RNA was isolated from denuded oocytes matured at 18 h using the RNeasy Micro Kit (Qiagen, cat.No.74004). cDNA synthesis was performed using a QuantiTect Reverse Transcription Kit (Qiagen, cat.No.205311). Real-time qPCR analysis was conducted using StepOne Plus (Applied Biosystems). Reactions were performed technically in triplicate and were repeated biologically three times. The housekeeping gene *EF1A1* was used as the endogenous control. The primer sequences used were listed in Table 1 .

**Table 1:** Sequence information on porcine-specific primers for quantitative real-time polymerase chain reaction.

Gene	Primer sequence (5ˊ-3ˊ)	Product size (bp)	GenBank accession no.
BMP15	F: CGCCATCAACTTCACCTAGCT R: CAGCAGGGAAGGCTTTAAGG	120	NM_001005155.1
EF1α1	F: AATGCGGTGGGATCGACAAA R: CACGCTCACGTTCAGCCTTT	120	NM_001097418.1

Abbreviations: F, forward; R, reverse.

### Statistical analysis

All experiments were repeated at least three times. All data were expressed as mean ± standard error of mean (mean ± S.E.M) and SPSS (Version 17.0) was used to perform single factor analysis of variance (ANOVA) for the percentage of polar body extrusion, 2-cell, blastocyst, total cell number per blastocyst, *BMP15* expression, oocyte with cortical and peripheral sub-membranous distribution of cortical granules and oocyte with mitochondrial distribution in the cytoplasm. P < 0.05 was considered statistically significant.

## Results

### Human exhaled air maintains normal nuclear maturation of porcine oocytes

**Figure 1:**
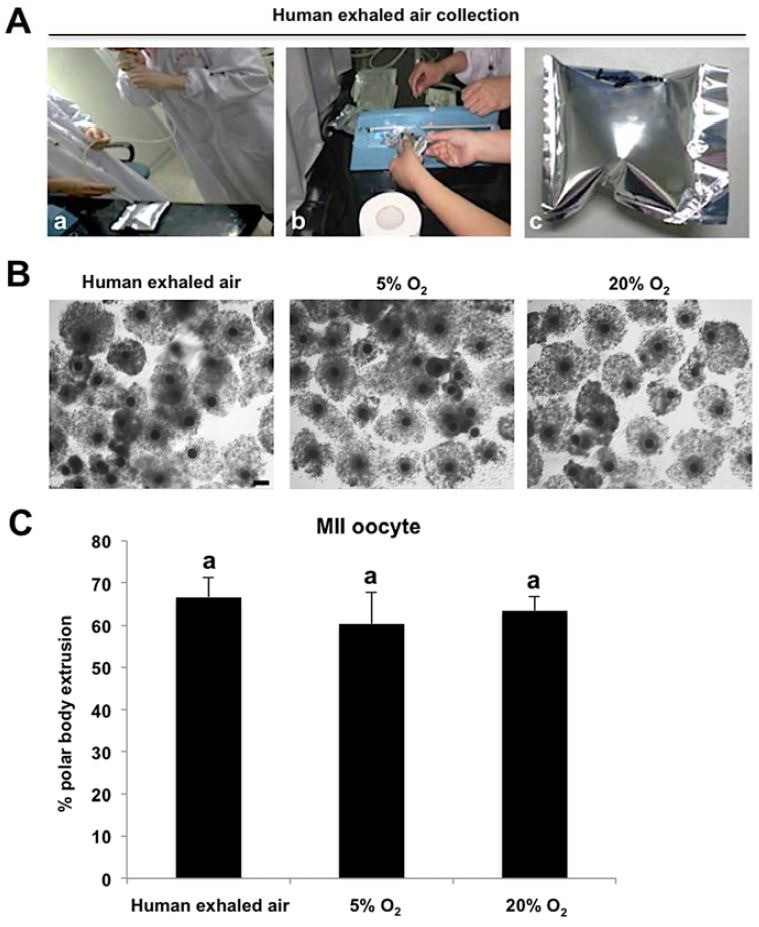
Human exhaled air maintains normal meiotic maturation of porcine oocytes. (A) The procedures of human exhaled air preparation. a. Porcine cumulus-oocyte complexes (COCs) at germinal vesicle (GV) stage were cultured in four-well plates containing *in vitro* maturation (IVM) medium covered with mineral oil. Four-well plates were transferred into the sterilized airtight aluminium bag and then experimenters exhaled air into the bag. b. Outside edge of bag should be sealed immediately when bag was inflated with appropriate human exhaled air. c. Shown was an aluminium bag inflated with human exhaled air. (B) Representative images of COCs with expanded cumulus cells matured *in vitro* in different air phases for 44 h. Scale bars: 100 µm. (C) Quantitative analysis of polar body extrusion (PBE) rate for COCs matured *in vitro* in different air phases for 44 h. The experiment was conducted four times with 240 GV oocytes per group. All the percentage data are expressed relative to the number of GV oocytes and shown as mean ± S.E.M. Values with different superscripts across groups indicate significant differences (P < 0.05). MII denotes metaphase stage of meiosis II.

To explore the effects of different oxygen tensions on the nuclear maturation *in vitro* of immature porcine oocytes, we cultured cumulus-oocytes complexes (COCs) for 44 h under different air phase conditions involving human exhaled air, 5% O_2_ or 20%O_2_ in air. Two air phases including 5% O_2_ and 20% O_2_ in air that are commonly used to culture oocytes were set as positive control. Assessment of nuclear maturation rate was indicated by the percentage of first polar body extrusion. At first, the whole procedures of human exhaled air preparation including three important steps were shown in Figure 1A . The peripheral compacted cumulus cells surrounding oocytes cultured in three air phases have significantly expanded and loosed after 44 h of culture and the expansion extent of the cumulus cells appeared to be comparable among different groups ( Fig. 1B ). Moreover, there was not significantly different in the rate of first polar body extrusion among three groups (67% versus 60%, 63%; Fig. 1C ). Therefore, these results indicate that human exhaled air is sufficient to support the meiotic maturation *in vitro* of porcine oocytes.

### Human exhaled air sustains robust distribution of cortical granules and mitochondria during porcine oocyte maturation

**Figure 2:**
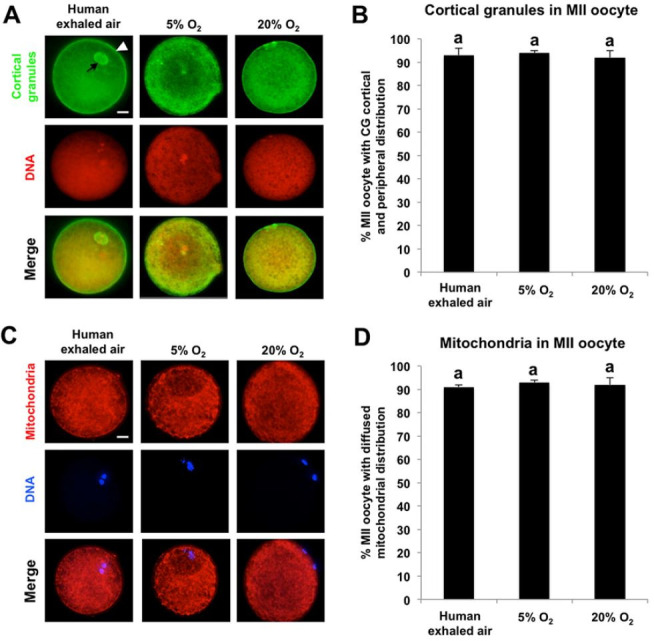
Human exhaled air sustains robust distribution of cortical granules and mitochondria during porcine oocyte maturation. (A) Immunofluorescence analysis of cortical granules (CG) with cortical area and peripheral sub-membranous distribution in denuded metaphase-II (MII) oocytes cultured in different air phases. Cortical granules and DNA in denuded MII oocytes were labeled with FITC-conjugated peanut agglutinin (green) and propidium iodide (red). Bottom panels showed the merged images (yellow) between cortical granules signals and DNA staining. Shown are representative Z-stacks obtained by epifluorescence microscopy from one experiment. Arrow indicates the first polar body, arrowhead denotes sub-membranous region. Scale bars: 20 µm. (B) Quantification of the percentage of MII oocytes with GC displaying cortical area and peripheral sub-membranous distribution in A. Data are shown as mean ± S.E.M. The experiment was repeated three times with 60 oocytes (human exhaled air), 64 oocytes (5% O_2_), 48 oocytes (20% O_ 2_), respectively. Values with different superscripts across groups indicate significant differences (P < 0.05). (C) Immunofluorescence analysis of active mitochondria with diffused distribution in denuded metaphase-II (MII) oocytes cultured in different air phases. Mitochondria and DNA were labeled with MitoTracker Red CMXRos (red) and Hoechst33342 (blue). Bottom panels showed the merged images between cortical granules signals and DNA staining. Shown are Z-stacks obtained by epifluorescence microscopy from a representative experiment. Scale bars: 20 µm. (D) Quantification of the percentage of MII oocytes exhibiting diffused mitochondrial distribution in C. Data are shown as mean ± S.E.M. The experiment was performed three times with 44 oocytes (human exhaled air), 56 oocytes (5% O_2_), 52 oocytes (20% O_2_), respectively. Values with different superscripts across groups indicate significant differences (P < 0.05).

The distribution patterns of cortical granules and mitochondria are usually used to assess the status of cytoplasmic maturation in mammalian oocytes after meiotic maturation ( Sha *et al.*, 2010 ). Furthermore, previous studies showed that migration of cortical granules (CGs) to the cortical area is a common hallmark in oocyte meiotic maturation ( Wessel *et al.*, 2002 ). Based on this information, we matured COCs in three air phases to metaphase II (MII) stage indicated by the first polar body extrusion and further analyzed the proportion of MII oocyte displaying cortical area and peripheral sub-membranous distribution. We found that CGs in the majority of MII oocytes matured in three air phases exhibited the distribution pattern of cortical and peripheral sub-membranous area ( Fig.2 A ). Besides, the percentage of MII oocytes displaying cortical and peripheral sub-membranous localization of CGs is similar among three groups ( Fig. 2B ). Since mitochondrial distribution in cortical area and inner region of oocyte cytoplasm has been observed in matured *in vitro* porcine MII oocytes ( Yang *et al*., 2010 ), we only here examined mitochondria with the certain distribution pattern in MII oocytes matured under different air phase conditions. Interestingly, we observed that mitochondria in most of MII oocytes matured in three air phases evenly diffused throughout the whole cytoplasm and cortical area ( Fig. 2C ). Moreover, there was no difference in the percentage of MII oocytes showing the cytoplasmic and cortical area distribution of mitochondria among three groups ( Fig. 2D ). Altogether, these results indicate that human exhaled air can maintain the normal distribution patterns of CGs and mitochondria to ensure the robust cytoplasmic maturation of porcine oocytes.

### Human exhaled air maintains correct expression of BMP15 essential for porcine oocyte maturation

**Figure 3:**
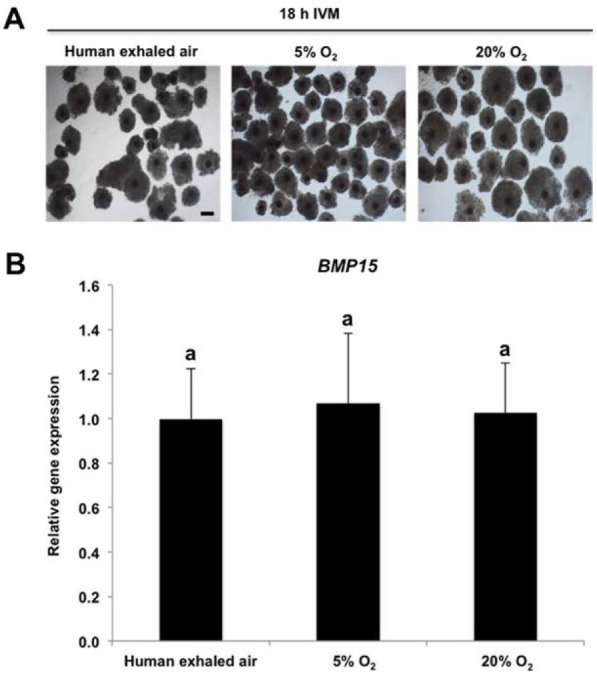
Human exhaled air maintains correct expression of BMP15 essential for porcine oocyte maturation. (A) Representative images of COCs matured *in vitro* in different air phases for 18 h. Scale bars: 100 µm. (B) Real-time qPCR analysis of BMP15 transcripts in denuded oocytes derived from COCs of A. Expression levels were normalized against endogenous housekeeping gene EF1α1 and human exhaled air group was set to 1. A total of three biological replicates were performed. Data are shown as mean ± S.E.M. Values with different superscripts across groups indicate significant differences (P < 0.05). IVM denotes *in vitro* maturation.

Oocyte-secreted factor BMP15 is a critical regulator for cumulus expansion during oocyte maturation ( Buccione *et al.*, 1990 ) and the maximum expression abundance of *BMP15* gene was observed in porcine oocytes matured *in vitro* for 18 h ( Li *et al.*, 2008 ). To examine whether oocytes matured *in vitro* under different air phase conditions have normal expression levels of *BMP15*, we employed qPCR to detect the expression abundance of *BMP15* in porcine oocytes matured *in vitro* for 18 h ( Fig. 3A ). qPCR analysis showed that *BMP15* expression level was not statistically different among three groups ( Fig. 3B ). Hence, our results indicate that human exhaled air does not alter the expression abundance of *BMP15* required for porcine oocyte maturation.

### Oocytes matured in human exhaled air support the early development of parthenogenetic activated embryos

**Figure 4:**
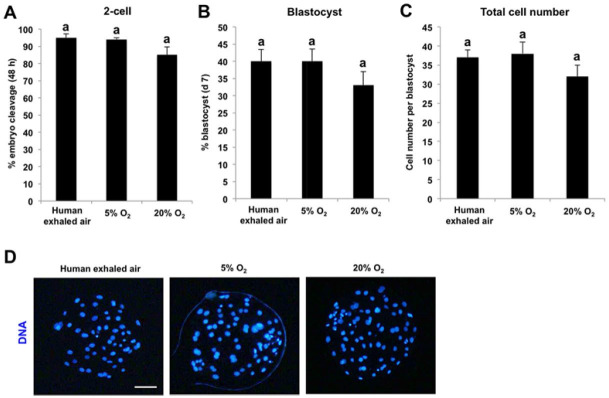
Oocytes matured in human exhaled air support the early development of parthenogenetic activated embryos. Metaphase II (MII) oocytes matured *in vitro* in different air phases were parthenogenetically activated (PA) by electric pulse. PA embryos were then cultured for 7 days in fresh PZM-3 medium at 38.5°C and 5% CO_ 2_ in humidified air. The cleavage rate (48 h) (A), blastocyst rate (day 7) (B) and total cell number per blastocyst (C) were statistically analyzed. Data are shown as mean ± S.E.M. The experiment was repeated four times with 240 embryos per group. Values with different superscripts across groups indicate significant differences (P < 0.05). (D) Representative images of PA blastocysts derived from MII oocytes matured *in vitro* in different air phases. DNA was labeled with Hoechst33342 (blue). Scale bars: 100 µm.

Given that oocytes matured in human exhaled air have normal nuclear and cytoplasmic maturation, we want to explore whether the resulting oocytes cultured in human exhaled air support the early development of parthenogenetic activated (PA) embryos. MII oocytes matured *in vitro* under different air phase conditions were parthenogenetically activated and cultured for 7 days in a humidified incubator at 38.5°C and 5% CO_2_. There was no significant difference in the cleavage rate, blastocyst rate and total cell number per blastocyst among groups ( Fig. 4A, B, C, D ). Therefore, these data demonstrate that oocytes matured in human exhaled air are able to support the early development of parthenogenetic activated embryos.

### Oocytes matured in human exhaled air support the early development of somatic cloned embryos

**Figure 5:**
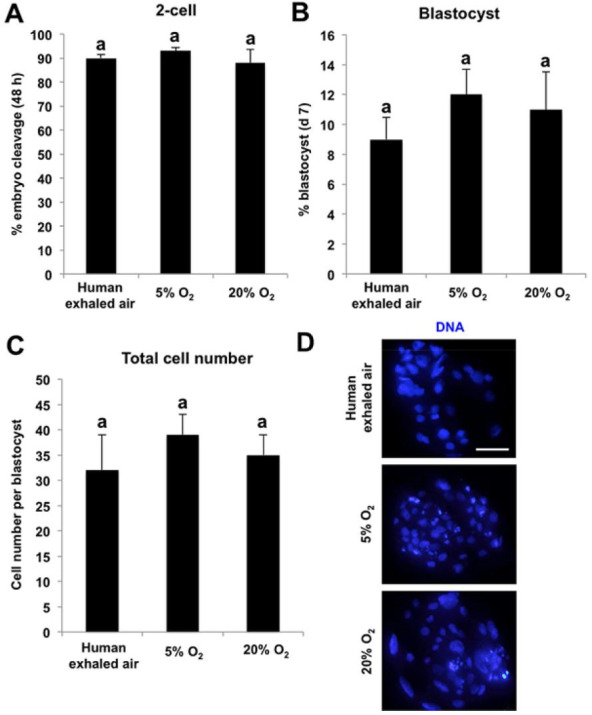
Oocytes matured in human exhaled air support the early development of somatic cloned embryos. Somatic cell nuclear transfer (SCNT) embryos were generated though transplanting donor cells into enucleated MII oocytes matured in vitro in different air phases. SCNT embryos were then cultured in fresh PZM-3 medium at 38.5°C and 5% CO_2_ in humidified air for 7 days. The cleavage rate (48 h) (A), blastocyst rate (day 7) (B) and total cell number per blastocyst (C) were statistically analyzed. Data are shown as mean ± S.E.M. The experiment was repeated four times with 120 embryos per group. Values with different superscripts across groups indicate significant differences (P < 0.05). (D) Representative images of SCNT blastocysts derived from MII oocytes matured in vitro in different air phases. DNA was labeled with Hoechst33342 (blue). Scale bars: 100 µm.

To investigate whether enucleated oocytes matured in human exhaled air can reprogram the terminally differentiated somatic cells to the totipotent state, we examined the early developmental competency of somatic cloned embryos derived from oocytes matured under different air phase conditions. There was no significant difference in the cleavage rate, blastocyst rate and total cell number per blastocyst among groups ( Fig. 5A, B, C, D ). Therefore, these data suggest that oocytes matured in human exhaled air are able to successfully perform the reprogramming events to ensure the normal early development of somatic cloned embryos.

## Discussion

In the present study we discovered that human exhaled air can efficiently support the nuclear and cytoplasmic maturation of porcine oocytes from which parthenogenetic activated and somatic cloned embryos derived have comparable developmental efficiency and quality with two traditional air phases ( Kang *et al*., 2012 ). This incubation system is an inexpensive, portable and reliable approach that is alternative to the traditional large gas-filled incubator and external gas tank. Therefore, it is very suitable to culture oocytes in the remote labs and animal farms, and even acts as a back-up gas supply apparatus to the current labs.

Previous study indicated that oxygen concentration is the main different composition among three air phases in which oxygen level in human exhaled air parallels to 20% O_2_ in air, but higher than 5% O_2_ in air (Vajta, 1997; Wang and Sun, 2007 ). Oxygen gas is thought to be an indispensable environmental factor influencing oocyte maturation. Furthermore, low oxygen tension *in vitro* paralleling to the physiological oxygen level in animal reproductive tract could be benefit to the oocyte maturation ( Iwamoto *et al*., 2005 ). However, we did not find the analogous phenomenon in our study because the rate of meiotic maturation is similar among human exhaled air, 5% O_2_ and 20% O_2_ in air. Consistent with our results, low oxygen concentration during *in vitro* maturation (IVM) has no significant effects on nuclear maturation of porcine oocytes ( Park *et al*., 2005 ). This discrepancy could be due to the diverse maturation mediums or protocols used in the different labs. Anyway, human exhaled air used in the simple incubation system does not at least exert the visible adverse effects on nuclear maturation of porcine oocytes compared to other two traditional air phases.

To further examine whether human exhaled air affects the cytoplasmic maturation of porcine oocytes, we assessed the distribution patterns of cortical granules (CG) and mitochondria in metaphase II (MII) stage oocytes and expression levels of *BMP15* in oocytes matured at 18 h. It is well known that CGs migration and mitochondrial distribution are two clear hallmarks of oocyte cytoplasmic maturation for many species ( Wessel *et al*., 2002 ). Previous studies showed that CGs migrate to the cortical and sub-membranous area during porcine oocyte maturation ( Sha *et al*., 2010 ; Yang *et al*., 2010 ; Zhang *et al.,* 2010 ). The disorganization of CGs would reduce the oocyte quality and even impaired subsequent embryo developmental competence ( Huan *et al*., 2015 ). Our results indicated that most of CGs migrated to cortical and sub-membranous area in MII oocytes matured in three air phases and no significant difference was also observed for the percentage of MII oocytes with cortical localization of CGs. The relocation pattern of CGs in porcine MII oocytes in our study is consistent with the data in other study in which porcine oocytes were incubated in traditional air phase ( Sha *et al*., 2010 ). Oocytes need to obtain the robust activity and localization of mitochondria to ensure the successful completion of oocyte cytoplasmic maturation ( Van Blerkom and Runner, 1984 ). Indeed, whether or not correct relocation of mitochondria is tightly related with the developmental competence of oocytes ( Brevini *et al*., 2005 ; Brevini *et al*., 2007 ). It is reported that mitochondria should translocate from periphery area at GV stage to the inner cytoplasmic regions at MII stage during porcine oocyte maturation both *in vitro* ( Sun *et al*., 2001 ) and *in vivo* ( Torner *et al*., 2004 ). In line with this data, in our study the mitochondria in the majority of MII oocytes matured in three air phases uniformly diffused in the cytoplasm. Therefore, human exhaled air could maintain the correct distribution patterns of CGs and mitochondria to ensure the acquisition of sufficient cytoplasmic maturation.

BMP15 is an oocyte-secreted factor important for oogenesis and folliculogenesis in mice ( Yan *et al.*, 2001 ). In sheep, BMP15 is reported to be critical for ovulation and fertility ( McNatty *et al.*, 2005 ). In pig, dynamic expression of *BMP15* during oocyte maturation has been characterized. Specifically, *BMP15* not only initiates its expression, but also reaches to the maximum level at 18 h of IVM culture, which is consistent with the beginning of cumulus expansion ( Li *et al*., 2008 ). Therefore, BMP15 may be important for cumulus cell expansion during porcine oocyte maturation. In our study, there was no significant difference in *BMP15* expression levels in oocytes matured *in vitro* for 18 h among three air phases. This imply that human exhaled air could maintain normal *BMP15* expression compared to other two air phases.

Maternally inherited RNA and proteins during oocyte growth and maturation is considered important for reprogramming of sperm or somatic cells to totipotent embryos ( Miyamoto *et al*., 2011 ; Zheng *et al*., 2013 ; Kong *et al*., 2014 ). Especially, cytoplasmic maturation involving appropriate synthesis of maternal RNA is necessary for embryonic genome activation and blastocyst formation ( Chen *et al*., 2013 ). Thus far, the relatively accurate method to assess cytoplasmic maturation status is tracking the developmental competence of early embryos ( Krisher, 2004 ). Although cytoplasmic maturation of porcine oocytes matured in human exhaled air appeared to be robust, as indicated by the cellular features of CGs and mitochondria distribution, whether these matured oocytes can support the early development of embryos derived from parthenogenetic activation (PA) and somatic cell nuclear transfer (SCNT) need to be confirmed. We found here that the cleavage rate, blastocyst rate and total cell number per blastocyst did not differ among different groups. These results further confirmed that porcine oocytes matured in human exhaled air successfully perform cytoplasmic maturation during IVM, which is sufficient to sustain the early development of PA and SCNT embryos. Further researches are required to classify whether these matured oocytes cultured under human exhaled air condition can produce somatic cloned piglets.

In conclusion, human exhaled air is a simple, cost-effective, portable and reliable atmospheric phase that can efficiently support *in vitro* maturation of porcine oocytes and subsequent early embryonic development.
